# Global Nationalism in Times of the COVID-19 Pandemic

**DOI:** 10.1017/nps.2020.35

**Published:** 2020-04-27

**Authors:** Florian Bieber

**Affiliations:** Centre for Southeast European Studies, University of Graz

**Keywords:** Nationalism, pandemic, authoritarianism, disease

## Abstract

The article outlines the impact of the COVID-19 pandemic on nationalism around the world. Starting from the premise that nationalism is a global and ubiquitous idea in the contemporary world, it explores whether exclusionary tendencies have been reinforced by the pandemic. The pandemic and government responses will not necessarily trigger the increase in exclusionary nationalism that both far-right politicians and observers have noted. However, there are 4 aspects, examined in the article, that might be shaped by the pandemic. These include the recent trajectory of nationalism and its social relevance prior to the pandemic,the rise of authoritarianism as governments suspend or reduce democratic freedoms and civil liberties, the rise of biases against some groups associated with the pandemic, the rise of borders and deglobalization, and the politics of fear. Thus, while the rise of exclusionary nationalism might not be the inevitable consequence of the pandemic, it risks reinforcing preexisting nationalist dynamics.

*We are all nationalists now.*

—*Nigel Farage (2020)*

The global pandemic and the response by governments in most countries has created an emergency felt on a scale like few other events in human history. Even the two World Wars were far from being global, with large parts of the world, from Latin America to most of Sub-Saharan Africa, only marginally affected. The near-simultaneous lockdown of a substantial share of the world’s population is a unique shared experience. Foremost, it is public health concern for millions, but both the disease itself and the response of governments and societies has deeply shaped and will continue to shape the world. While the pandemic lasts and the human loss, the economic collapse, the closure of borders, and other effects are felt, it is easy to imagine that the post-pandemic world will be fundamentally different than the one that came before. However, change and continuity are always closely intertwined in the transformation of human society. So, while the after will be different, it will be built on the before.

As the world seemed to come to a standstill, a number of observers have noted that a global rise of nationalism might be a consequence of the pandemic and states’ responses (Rachman 2020; Tisdall 2020; Harari 2020). From the closure of borders and the difficulty of mobilizing support and solidarity across them, to the fear many people experience, the response appears to make nationalism more salient as people look to support their own communities.

However, the opposite argument could also be made. The shared global experience, and the fact that the “enemy” is not another nation or group but an indiscriminate virus, might promote greater cross-national solidarity and cooperation. The inability to confront the pandemic within the confines of the nation-state underlines the need for cooperation, even if the immediate response has often been otherwise.

It is far from clear whether the world will be confronted with what some have called “coronationalism” or global solidarity (Ozkirimli 2020). Forcasting the post-pandemic world is both tempting and difficult. Initial responses, as well as the environment of crisis, might prove to be misleading or temporary indicators rather than reliable predictors. However, nationalism has become visible in numerous forms during the pandemic response. Considering the increased importance of exclusionary nationalism in numerous societies and political debates around the world prior to the pandemic, this is no surprise. It thus merits reflection on how both the pandemic itself and the various government responses are likely to shape the role of nationalism in the world.

Nationalism has become a central ideology and practice around the world over the past two centuries. It is a central feature of contemporary society, from the structure of the state system into units that commonly define themselves as nation–states, to the potency of national movements to affirm states or challenge them (Malešević 2019). Nationalism is best understood to be a malleable and narrow ideology that values membership in a nation more than belonging to other groups, and it gives preference to political representation by the nation for the nation (Bieber 2020a, 10). In brief, nationalism assumes the dominant political and social importance of the nation and understands the world to be structured in nations, often with the understanding that these are the natural units of social and political organization. It is thus perhaps obvious to assume that the global pandemic will impact this structural feature of the contemporary world. As nationalism is ubiquitous and takes form in a variety of ways, from individual biases and political parties to foreign affairs and government policy, there is no single effect or trajectory that can be mapped with any certainty. In particular, in the middle of the pandemic, it is impossible to give a clear assessment of how the pandemic and government responses will shape nationalism. This contribution is thus cautious and inherently preliminary, a first draft at best.

## Disasters, Disease, and Nationalism

The interplay of pandemics, or even natural disasters more broadly, and nationalism has received little attention in the academic literature. This is no surprise, as there have been few global pandemics since the rise of the modern nation, and research on public health and disease and social and political ideas such as nationalism are rarely interlinked. Ironically, the language of discussing nationalism and pandemics is similar. The notions of spread and contagion, terms borrowed from the realm of diseases, are often applied to the study of nationalism. The link was made explicit by Albert Einstein, who claimed that “nationalism is an infantile disease. It is the measles of mankind,” (1929). Of course, this linguistic link is not necessarily helpful for understanding the dynamics between the two.

The experience of the 1918–20 Spanish flu^1^ is of limited help. The pandemic began in the last year of World War I and killed between 17 and 100 million people around the world within two years. Its peak during such events as the end of World War I, the Russian revolution, and the breakup of major empires results in it being overshadowed by these events, even if the experience of many ordinary citizens at the time may have been more profoundly shaped by the pandemic. In many ways, the pandemic contributed to the larger trauma of the war and compounded the collapse of the pre-war era, which in Europe was associated with the stability of the Great Power balance and elsewhere with the experience of colonialism. Thus, it is impossible to disentangle the impact of the influenza pandemic on nationalism. As Laura Spinney outlines in her masterful study of the Spanish flu and its consequences, rather than one outcome, it contributed more broadly in shaping the emergence of public healthcare and international institutions, and it impacted local politics across the world: different mortality rates and the impact on important individuals, as well as soldiers and civilians in the last months of the war, shaped both the end of the war and the peace that followed (2017).

Disease, like other natural disasters, is first and foremost an exogenous shock to society that can trigger greater cooperation (Kelman 2011), such as the 1999 Greek-Turkish earthquake diplomacy (Ker-Lindsay 2000). Other cases, such as droughts, can exacerbate tensions and conflict.^2^ Natural disasters can be understood as critical junctures or moments of uncertainty, often induced by exogenous and indigenous crises. They are critical because the institutional and policy paths that are chosen at such moments are difficult to change in normal circumstances (Capoccia and Kelemen 2007, 341–342). This historical institutionalist argument does not imply historical determinism but distinguishes between moments of greater opportunity for large-scale change and periods where such choices are rare and unusual. Critical junctures do not imply that a radical change is the inevitable outcome but rather that it becomes a greater possibility. Furthermore, such crises do not imply any particular outcome. A pandemic, a natural disaster, a major economic crisis, a war, or a state collapse—each constitutes such a critical juncture (i.e., they are both rare and constitute a major disruption). The concept of critical junctures is commonly employed to understand institutional change but could also be evoked to study larger social transformation.

To understand the outcomes that could emerge as a result of the pandemic and societal and state responses to it, and their impact on nationalism, one can consider the following aspects that provide the context and the first effects of the pandemic: (1) the recent trajectory of nationalism and the social relevance of nationalism prior to the pandemic; (2) the interrelationship between authoritarianism and nationalism and the impact of the pandemic on this linkage; (3) how the pandemic is shaping biases; (4) the (temporary) deglobalization and the closure of borders; and (5) the role of fear. These five dimensions allow for some preliminary considerations of how nationalism is being shaped by the pandemic and responses to it.

### Recent Trajectory of Nationalism

Nationalism is universal to the extent that it is nearly impossible to determine its strength or fluctuation on a global level. It is furthermore empirically difficult to capture, as the concept of nationalism cannot be easily grasped through one idea or definition. Recent years have seen an increase of important political leaders and parties promoting an exclusionary nationalist policy. These are expressed in a variety of forms, such as the “America First” platform of US president Donald Trump, the Vote Leave campaign for Brexit, the success of the Hindu nationalism of Narendra Modi, the nationalism promoted by Benjamin Netanyahu in Israel, and the conservative nationalism of Japanese Prime Minister Shinzō Abe. In addition, populist and far-right parties have been successful in numerous European elections and at times joined ruling coalitions and shaped government policies (Bieber 2018).

There is considerable overlap between what I have termed exclusionary nationalism (Bieber 2020a, 15) with the political far right, namely, political parties that seek to establish ethnocracies (i.e., monoethnic states dominated by a specific nation that is usually defined in narrow ethnic terms [Mudde 2019, 64]). Cas Mudde (2019, 50–56) has convincingly argued that the far right has entered the political mainstream after 2001, which in addition to their increase in electoral support contributes essentially to their significance.

Exclusionary nationalism can express itself in political organizations, from parties (or parts of parties) to media and civil society. It can also be reflected in widely held attitudes and their public expression. The success of exclusionary nationalist parties and candidates is at times only marginally the result of nationalism itself. Instead, such political actors draw on other themes, such as populist strategies, namely, claiming to represent the majority against an alien elite or using corruption and other themes to gain success. However, whether on the way to power or after they assume it, exclusionary nationalists shift the agenda and change the acceptable public discourse. Particularly common themes have been the supposed threat posed by migration and Islam, a discourse that increased in potency, especially in Europe, in the aftermath of the so-called migration crisis. However, anti-migrant and anti-Muslim themes can be found in exclusionary nationalist discourse elsewhere, including Myanmar, India, and the USA.

The rise and electoral success of far-right parties and candidates has led to a shift of the public debate, often pursued by these actors (Sharpe 2017; Robertson 2018). Importantly, the rise of the far right and their political mainstreaming has meant that exclusionary nationalist ideas have become more widespread and socially accepted in numerous countries. These discursive and social shifts are more enduring than the policies or rhetoric of any given party or official. Policies and public debates on migration and Islam in most European countries best illustrate how exclusionary nationalist parties and movements have changed the political agenda.

This does not mean that this shift is universally shared or permanent. In fact, exclusionary nationalism is highly polarizing and divisive as it is often combined with populism, which denies the legitimacy of alternative political positions. For example, in the USA, the most important policy issues for self-declared Republicans and Republican-leaning independents in January 2020 were terrorism, the economy, and immigration, none of which figured in the top spots for self-declared Democrats or Democratic-leaning independents (Pew Research Centre 2020).

Closely linked to the shift in public debate are both state policies and social behavior. Migration policies have become more repressive (Wallace and Young 2018; Chin 2017). Recent years have also seen significant increases in hate crimes and discrimination. The OSCE (Organization for Security and Cooperation in Europe) has been recording such data among its member states, and while the data is not comparable across countries due to different methodologies and context, there is a marked increase in key countries. For example, in Germany and the UK hate crimes reported by the police more than doubled between 2014 and 2018; in other countries like the USA, it increased substantially (OSCE ODIHR 2019).^3^ The increase appears to be linked to the shift in public discourse and crucial events such the election of Donald Trump in the USA, the Brexit referendum in the UK, and so-called migration crisis in Europe, which all occurred between 2015 and 2016 (Edwards and Rushin 2018; Cuerden and Rogers 2017; Rees et al. 2019).

Thus, the COVID pandemic began against a backdrop of strong mainstreamed exclusionary nationalism in key countries around the world, in particular in Europe and North America. The pandemic has overshadowed these earlier issues, but it remains uncertain whether anti-immigrant and anti-Muslim policies and discourse have been permanently eclipsed (i.e., whether they will reemerge after the end of the pandemic emergency). Furthermore, the pandemic may also reinforce some of these anti-immigrant and isolationist policies and positions.

### Nationalism, Authoritarianism, and the State of Emergency

Exclusionary nationalism and authoritarianism often enjoy a symbiotic relationship. Of course, there are political parties and other actors that are nationalist and not authoritarian, and there are autocrats who derive their legitimacy from sources other than nationalism. However, the combination is common, as exclusionary nationalism has strong authoritarian themes, such as the importance of the collective nation over individuals and its exclusionary and Manichean understanding of politics. For autocrats, nationalism is often a ready-made ideology that does not challenge their rule and can be used to mobilize against challengers.

Finally, there has been an increase of authoritarian and populist regimes concurrent with increased nationalism globally. It is thus appropriate to argue that authoritarianism, populism, and exclusionary nationalism have been closely interlinked and often mutually reinforcing (Bonikowski 2017; Jenne 2018).

In an early reflection on the impact of the pandemic on global politics, Ivan Krastev noted the return of experts to the forefront of discourse. In brief, populism and authoritarianism have in recent years discredited the importance of experts and relativized the notion of truth. The notorious claim by President Trump’s advisor Kellyanne Conway about “alternative facts” suggested that facts are not established or objective but subject to political claims and counter claims. Krastev noted that “professionalism is back in fashion,” (2020). As nonexpert instructions to ignore the pandemic or rely on herd immunity failed to contain its spread and increased the risk to human life, expertise by professionals has gained ground. When facts are not about the number of attendees at a presidential inauguration but rather the life or death of individuals, including friends and relatives, expertise becomes more important. This shift would also imply that populist and far-right parties would stand to lose from such a dynamic, as it is these parties that have openly questioned the importance of expertise and supported conspiracy theories. In some countries, polling data seems to bear out such trends. For example, in Germany the ruling coalition, and in particular Chancellor Angela Merkel, has gained popularity since the onset of the crisis, while the far-right Alternative for Germany (AfD) has lost political ground, dropping to around 10 percent of electoral support in late March 2020, its lowest rating in over two years. There is, however, no wider European or global trend. In Italy, where the far-right Lega and Fratelli d’Italia are in opposition, both have been holding steady at more than 40 percent support since 2019. Patterns of decline in electoral support often preceded the coronavirus crisis, as in Austria and Germany (*Politico* 2020). It is true that during the crisis not only has the importance of expertise increased but also that the classic topics of the far right, such as migration and Islam, have been sidelined. However, it would be simplistic to assume that the pandemic would threaten populism or the far right. While some populists have responded late and erratically to the crisis, such as US President Trump, British Prime Minister Boris Johnson, and Brazilian President Jair Bolsonaro, others, including Israeli Prime Minister Benjamin Netanyahu and India Prime Minister Narendra Modi, have responded with strong measures. In both cases the responses have been self-serving and framed in the rhetorical style of the incumbent (Mudde 2020).

Thus, it would be overly optimistic to assume that the pandemic will shift the focus away from the classical themes of exclusionary nationalists and that a new era of expertise driven politics is likely to emerge. The pandemic has allowed for an unprecedented restriction of civil liberties and freedoms across the world, in both established democracies as well as in authoritarian regimes. Hungarian Prime Minister Viktor Orbán has used the crisis to increase the extent of his political control by sidelining parliament and ruling by decree (Hopkins and Hall 2020).

Emergency politics are a central theme of authoritarian rule. Emergencies have given autocratic leaders the opportunity to destroy or suspend democratic institutions and their checks and balances. The most prominent historical example is the Reichstag fire in February 1933. Occurring during the early weeks of Nazi rule, it allowed the government led by Adolf Hitler to pass the sweeping Enabling Act (*Ermächtigungsgesetz*) that provided the basis for his unchecked rule. Emergencies are often moments when the executive gains power, and due process is suspended (Snyder 2017). This leads to what Johnathan White has called “*emergency politics*, in which actions departing from conventional practice are rationalised as necessary responses to exceptional and urgent threats” (2015, 300; italics in the original). There is little doubt that the threat of the pandemic justified measures such as postponing elections (i.e., the Democratic primaries in the USA, parliamentary elections in Serbia and North Macedonia), the assembly of large groups of people, and the closure of some institutions. However, the risk from such derogations is twofold. First, the measures have to be proportional to the desired goal, and the judgment of proportionality can be difficult in vibrant democracies, not to mention weaker democracies and competitive authoritarian regimes. Second, autocrats can seize this opportunity to increase their control and dismantle checks and balances (Bieber 2020b).

While authoritarianism could thrive due to the global pandemic, nationalism need not follow the same trajectory. In fact, populists and autocrats might shift from the threat of migrants, terrorism, or Islam toward using the pandemic as the primary device to justify illiberal and undemocratic policies. However, such a disassociation might be temporary. Parties and governments that built their electoral success on such items are likely to maintain exclusionary nationalism as part of their platform and addend it with new themes. Furthermore, the pandemic will not provide an unlimited legitimation for a state of emergency. To continue with emergency powers, whether de facto or de jure, other sources of legitimacy might be needed.

As discussed in the next section, some populists and autocrats have blamed minorities or migrants and other so-called outsiders, creating a link between the disease and specific population groups. For example, in Hungary, Viktor Orbán blamed the opposition for the spread of the virus after it opposed a sweeping emergency bill, showing how the pandemic can easily be politically weaponized.

### Disease, Nationalism, and Bias: The “Kung Flu”

An iconic image of the global pandemic was a close-up of the daily press briefing of US president Donald Trump, captured by *Washington Post* photographer Jabin Botsford, that showed the statement to be read by the president with only the word *Corona* crossed out and replaced with *Chinese* (CNN 2020). The Trump administration has adopted the term *Chinese virus* to link the pandemic to China and also externalize responsibility for the spread. The president and high-ranking officials have defended the use of the term (Rogers, Jakes, and Swanson 2020).

Since the spread of COVID-19, there have been reports of increased racism and discrimination directed against individuals of Chinese origin or those who are assumed to be Chinese in the United States and beyond. Just within two weeks (March 19–April 1, 2020), an online reporting tool in the USA recorded 1,135 cases of COVID-19-related discrimination. Most cases involved verbal assault; others report being denied services, including transport, and being spat on and physically assaulted, usually with direct or indirect reference to COVID-19 (Jeung 2020). While anti-Chinese and anti-Asian bias has been particularly pronounced in the USA, facilitated by the discursive link made by President Trump, it has been noted elsewhere, including Europe and Australia (Escobar 2020; FRA 2020). This feeds into existing anti-Chinese and anti-Asian bias that has a long historical record. It has been fueled recently by the rise of China as a global actor and the increasing rivalry between China, the EU, and the USA.

The implicit and explicit link between COVID-19 and the Other has not just been directed against Chinese or Asians; other groups have been blamed for spreading the disease (OHCHR 2020). Hungarian prime minister Viktor Orbán claimed that the spread of the virus was linked to immigration, a central theme of his rhetoric since 2014. Without providing any evidence of the link, which appeared implausible, his government closed down the already highly restrictive asylum system (Inotai 2020). In the USA, the emergency rules to combat the pandemic have empowered the Department of Homeland Security to return illegal and undocumented migrants to their countries of origin without due process (Castellanos-Jankiewicz 2020). In India, officials of the ruling Bharatiya Janata Party likened Muslims to suicide bombers for protesting against the new citizenship law, as the government ordered a lock down and the ruling party and media have singled out Muslims as “supercarriers” (Daragahi 2020; Kazmin, White, and Palma 2020). Far-right parties in Europe, such as the Alternative for Germany (AfD) and the Austrian Freedom Party (FPÖ), have also linked the pandemic to the supposed threat of migration or have demanded repressive measures specifically aimed at migrants (Jansen 2020). In Central Europe, Roma became targets of discrimination, being blamed for spreading the disease (FRA 2020).

There is a long-established pattern of linking minorities, racial groups, and specific communities to disease. Exclusionary nationalism and racism often equate specific groups with diseases themselves. A prominent example is the anti-semitic discourse in Nazi Germany, which likened Jews to a disease effecting the body of the German nation. These notions of disease not only dehumanized people belonging to particular groups but also allowed for the promotion of an organic understanding of the nation that equated nations with living bodies. Considering people as parasites or diseases played a crucial role in normalizing their nonhuman status and justifying their murder.

Furthermore, minorities and other marginalized groups have often been described as carriers of disease, with their marginalization justified through the lens of public hygiene and controlling the spread of disease. In the Nazi Propaganda film *Der Ewige Jude* (The Eternal Jew, 1940), for example, Jews are compared to rats as carriers of the plague (Friedman and Koch 1989). From the 1892 cholera outbreak to HIV/AIDS and Ebola, minorities and vulnerable communities are easily made scapegoats for pandemics and epidemics (Bartholomew 2020). Blaming migrants or minorities for disease is well-established and risks remaining a strong line of discourse even if the pandemic itself recedes.

These links are also activated as implicit biases. A 2009 study during the swine flu epidemic suggested that those who had been vaccinated expressed fewer negative views about migration, as “their sense of vulnerability to disease was tied to unacknowledged fears about infected immigrants” (Eberhardt 2019). Such findings would suggest that the traumatic experience of the pandemic might have a long-term impact on implicit biases towards migrants.

At times, the response to the pandemic has resulted in disadvantaging marginalized groups and asserting the dominance of the majority. There are banal examples, from not providing warnings and guidelines in minority languages, to the case of Denmark, where naturalization was temporarily stopped with the outbreak of the pandemic, as the naturalization law requires a handshake with the official granting citizenship. The 2019 law sought to force migrants who for religious reasons reject handshakes to do so. Thus, the assimilationist law resulted in a de facto full suspension of naturalization, as social distancing rules did not permit handshakes (Strittmatter 2020). Even without an explicit link between the pandemic and vulnerable groups, government responses can deliberately or inadvertently lead to discrimination and reinforce exclusion.^4^

### Deglobalization and Old Borders

One immediate impact has been the rise of what has been called “medical nationalism” (Youde 2020), the strong support for national medical priorities, irrespective of the potential external costs or effects. Due to shortages and domestic worries, the first response of numerous governments is to reduce international cooperation. While some of the early reports of the scramble for medical supplies have overstated the antagonistic and adversarial dimension, they nevertheless shape public perceptions.

Member state restrictions on the export of medical supplies constituted an early problem within the EU, undermining the common market and jeopardizing the notion of solidarity in the bloc. One of the first measures of the EU in light of the global crisis was to establish Union-wide rules requiring permits for the export of medical supplies in short supply. This effectively Europeanized the restrictions and reestablished intra-EU cooperation (Bayer et al. 2020). However, elsewhere, where such structures do not exist, the rigidity of national borders became more apparent.

Beyond medical supplies and the reemergence of borders, the EU has been shaken by the national responses to the pandemic. Lacking competences in the field of public health, the EU had been institutionally ill equipped to respond. In particular, in countries strongly affected by the pandemic, such as Italy, the slow and halting response from other EU member states undermined their confidence in European solidarity. Such trends have been exploited by Eurosceptic parties, such as the far-right Lega in Italy, and reinforced the north-south divide in the EU dating back to the Eurozone crisis between 2010–2012 (Johnson, Fleming, and Chazan 2020).

Far-right and populist politicians from around the world have made temporary border closures in response to the pandemic, claiming vindication for their longstanding emphasis on closing borders. Most prominently, US president Donald Trump tweeted on March 23, “THIS IS WHY WE NEED BORDERS!” (Trump 2020). Laura Huhtasaari, a Member of the European Parliament of the far-right Finns Party*,* noted that that “the need for borders is being vindicated by the pandemic” (Kirschbaum, King, and and Bernhard 2020).

While far-right parties and political figures may be tempted to see their worldview vindicated by the global lock down, the link is not as straightforward. First, the closures are temporary. Even if they last longer than originally anticipated, they are neither intended nor could they realistically become permanent. Second, for the first time, many citizens in prosperous democracies in Europe and North America, used to easy mobility across borders, travel, and work abroad, are locked in, not just in their countries but at home. Whereas many citizens might support such measures as necessary steps during a crisis, it is implausible that this experience would generate support for such measures in the long term. The crisis has also highlighted the difficulties that closed borders entail. Furthermore, the spread of COVID-19 across borders has demonstrated that even highly repressive measures, including border closures and the collapse of international air traffic, could not stop the spread the disease. As a result, the limited utility of border controls during the pandemic might help to promote their reduction, rather than their maintenance, in the long term. In Europe, the aftermath of the so-called migration crisis resulted in the temporary introduction of border checks in multiple European countries and the gradual erosion of the Schengen Area system. However, as these checks mostly have affected migrants, with citizens from privileged countries largely exempted, many tolerated these restrictions. The pandemic induced restrictions, on the other hand, did not distinguish between citizens on the basis of wealth or prestige of the country of origin.

On a related note, the closing of borders around the world and evacuation of citizens by foreign ministries has reemphasized the importance of citizenship. Some of these expressions are primarily discursive. Austrian Chancellor Sebastian Kurz, in his statements to the public on the crisis, consistently addressed all Austrians, while ignoring the substantial number of permanent residents who are not Austrian citizens. The value of citizenship has shifted and increased, though it has not always followed traditional criteria, as its value has shifted based on which countries have been most affected. Initially, Chinese and Iranian citizens faced restrictions, but as the pandemic spread to Europe, it became Italians who encountered limits on travel. As borders reemerged across the continent and the world, citizenship enabled a return home but often placed foreigners in precarious positions.^5^ The restrictions introduced in February and March 2020 often did not distinguish between the place of residence or origin for travel and citizenship purposes. Thus, Chinese and other citizens became subjects of discrimination, even if they had not been traveling from so called hot spots (Dzankic and Piccoli 2020). As the pandemic has spread globally, the risk is that citizens from the Global South will be particularly affected by continuous border restrictions. The result could be a reinforcement of global inequalities of citizenship, especially as citizenship and migratory policies are likely to remain more rigid.

Certainly, the pandemic at first reinforced the primacy of the state (Rachman 2020; Krastev 2020). It is states that are providing security, ensuring the functioning of the health-care system, and intervening in the economy. The degree of state intervention that occurred within a few weeks of the outbreak of the pandemic across the world stood in stark contrast to role of the state in the neoliberal global system. In addition, reinforcing the role of the state has been pursued by governments across the ideological spectrum. The (temporary) rise of the state weakens the neoliberal paradigm that the market alone can regulate economic needs. In fact, states have intervened against free markets to secure supplies for their citizens. The primacy of the state also weakens global governance and cooperation. The EU, due to its relatively robust regime and rules, has been able to reassert itself after the initial crisis, but intra-EU solidarity has been brittle. As Gideon Rachman (2020) has argued, the pandemic has highlighted the vulnerability of individual countries to global supply changes, which in times of crisis and disruption leave many countries at risk of not receiving key medical supplies and other goods of critical importance. The retreat from globalization has been advocated and articulated by supporters of US president Donald Trump: “What is the point of pursuing internationalism even as an ideal, when interconnectedness itself exposes us to such serious risks? After all, the pandemic would have been far easier to manage if cross-border trade and travel were not so pervasive—if people, all along, had stuck closer to home,” (Waddy 2020). Whereas smaller countries are unlikely to overcome this global dependency, larger countries, such as the USA, could opt for greater protectionism. This would reinforce preexisting rivalries and strengthen nationalism and power competition.

The temporary deglobalization has been unprecedented, and the reopening of borders and the resumption of global traffic could take months, if not years. In the process of opening borders and resuming flows, considerations other than disease control will inform the de-restriction. In particular, populist and nationalist elites will, as they have in the first period the pandemic, combine disease control with nationalist and isolationist motives. While the closure of borders and national isolation is presumably temporary, some measures will be extended for a variety of motivations, often closely intertwined with exclusionary nationalism, and anti-globalization arguments will be reinforced.

### Politics of Conspiracy and Fear

An essential feature of the global pandemic has been the proliferation of fear. As *New York Times* correspondent Steven Erlanger aptly noted, “The coronavirus has created its own form of terror. It has upended daily life, paralyzed the economy and divided people one from another. It has engendered fear of the stranger, of the unknown and unseen,” (2020). Fear of illness or the hardship associated with government responses and the economic crisis are real and tangible for a substantial share of the global population. Fear is also a potent and dangerous motivator in political choices.

Extremist groups have used the pandemic as an opportunity to spread fear and conspiracy theories. The crisis has been weaponized in various forms to diverse ends: denying its importance and favoring various conspiracy theories; blaming particular racial or ethnic groups for the pandemic; and pushing for chaos and anarchy to promote a new political order (Colborne 2020). However, such groups, usually on the neo-Nazi spectrum of the far right, are politically marginal and often relish in apocalyptic visions. The pandemic has also given fuel to crude conspiracy theories, spread by social media. From the idea that 5G technology spread the virus, to classical conspiracy theories that the disease is merely a smokescreen for a government plot, there have been a wide range of bizarre ideas circulating since the beginning of the outbreak. These alleged conspiracies are spread by networks that have been linked to far-right and radical groups and activists. These include similar networks as the “Pizzagate” conspiracy in the USA (Robb 2017) and the so-called Reichsbürger in Germany (i.e., self-identified citizens of the German Reich, who believe that the current German Federal Republic is illegal; see Speit [2017]). Social media networks like 4chan and 8chan have provided space for unfiltered conspiracy theories, memes, and transgressions that have been seized upon by far-right groups and individuals over the past decade (Nagle 2017). Although small and relatively fringe groups, the ideas have circulated widely and been given prominence by far-right and populist politicians and parties. Conspiracy theories can undermine trust in democratic institutions and in extreme cases generate violence (Sunstein and Vermeule 2009), as religious rioting in India attests (Horowitz 2001, 74–77). In an environment of fear and uncertainty, conspiracy theories become particularly widespread and more likely to find a following. This feeds into some of the aforementioned risks of the pandemic and governments responses, namely, the scapegoating of minorities and the use of this context by far-right groups.

In addition to the fertile environment the pandemic provides for conspiracy theories, the psychological and economic effects of the pandemic will fuel the politics of fear. The extent of the economic crisis that individual countries and the global economy will face after the pandemic remains difficult to predict, but it will constitute one of the most serious economic crises of the past century (Giles 2020). Considering that the political consequences of the 2008–2009 economic crisis still reverberate, and the link between economic crisis and nationalism is well documented (Rantanen 2012), the global economic consequences of the pandemic are likely to fuel exclusionary nationalism.

## Nationalism in the Post-COVID-19 World

In “A World Less Open, Prosperous, and Free,” Stephen M. Walt, a key representative of the realist school of international relations, has argued, “the pandemic will strengthen the state and reinforce nationalism” (Allen et al. 2020). This view concurs with nationalist politicians themselves, who claim vindication for their position. However, such predications betray the biases of on observer like Walt or nationalist politicians themselves. A crisis is likely to be interpreted through the framework and ideological bias of each respective witness.

Nationalism is deeply engrained into the global system and most societies around the world. As such, the global coronavirus pandemic and state responses are not going to fundamentally alter this reality. Neither will the pandemic usher in a new era of global solidarity, nor is it likely to destroy the networks that globalization has created. As many citizens are locked in, including those from countries usually privileged in terms of their ability to cross borders, the appreciation of open borders may increase. Thus, a post-pandemic world with stronger borders seems unlikely. However, the opening of closed borders for all citizens will be a drawn-out process.

The fear created by the pandemic and the biases that have already been linked to COVID-19 are likely to be enduring, and they will shape the post-pandemic world. In the search for scapegoats, minorities and other vulnerable groups are likely to suffer and become targets of exclusion. In particular, migrants are vulnerable and have already been instrumentalized by far-right parties, characterized as carriers of disease. The past decade has been an era of anxiety, as crises, especially in Europe, have become common and normalized. The economic uncertainty after 2008, the narrative of migration as a threat, and now the pandemic all have contributed to a social environment of uncertainty and fear. In addition to the psychological consequences of collective anxiety, the political and social outcome is more likely to strengthen exclusionary nationalism.

Finally, government responses to the pandemic risk turning fragile democracies into competitive authoritarian regimes. Such competitive authoritarian regimes might initially rely on the pandemic to justify repressive policies, but they are likely to turn to exclusionary nationalism as a key legitimizing ideology in order to sustain power.

There is nothing inevitable about the dominance of exclusionary nationalism in the post-pandemic world, as some doomsayers, nationalists, and far-right figures proclaim. At a critical juncture, different paths are available, and the outcome will depend on the language used and the policy choices made during the crisis.

## Notes

1 It is named “Spanish” as Spain, being neutral in World War I, had less restrictive censorship than countries at war. As a result, the first reporting on the influenza pandemic began in Spain.

2 Although most scholarship is careful about the direct link between disaster and war, the causal link appears to be rather indirect Slettebak (2012).

3 In the USA, between 2014 and 2018 the increase of hate crimes recorded by police from rose from 6,385 to 8,496, in the UK from 52,853 to 111,076, in Germany from 3,059 to 8,113, and in France from 1,662 to 1,838.

4 In response to government measures, the OSCE High Commissioner on National Minorities has issued set of guidelines to ensure social cohesion and prevent discrimination (HCNM 2020).

5 A rare exception has been Portugal, which granted migrants and asylum seekers permanent residence (Euronews 2020). On the other hand, several countries with large numbers of migrants in affected areas, such as the Western Balkans, explicitly requested their own nationals not to return or identified them as particular carriers of the disease (Stojanovic 2020).

## References

[r1] Allen, John et al. 2020 “How the World Will Look After the Coronavirus Pandemic,” Foreign Policy, March 20, 2020. https://foreignpolicy.com/2020/03/20/world-order-after-coroanvirus-pandemic/. (Accessed April 9, 2020.)

[r2] Bartholomew, Robert. 2020 “The Coronavirus and the Search for Scapegoats.” Psychology Today, February 6, 2020. https://www.psychologytoday.com/us/blog/its-catching/202002/the-coronavirus-and-the-search-scapegoats. (Accessed April 9, 2020.)

[r3] Bayer, Lili, Jillian Deutsch, Jakob Hanke, and Paola Tamma. 2020 “EU Moves to Limit Exports of Medical Equipment Outside the Bloc.” Politico, March 15, 2020. https://www.politico.eu/article/coronavirus-eu-limit-exports-medical-equipment/. (Accessed April 9, 2020.)

[r4] Bieber, Florian. 2018 “Is Nationalism on the Rise? Assessing Global Trends.” Ethnopolitics 17 (5): 519–540.

[r5] Bieber, Florian. 2020a Debating Nationalism: The Global Spread of Nations. London: Bloomsbury.

[r6] Bieber, Florian. 2020b “Authoritarianism in the Time of the Coronavirus.” Foreign Policy, March 30, 2020. https://foreignpolicy.com/2020/03/30/authoritarianism-coronavirus-lockdown-pandemic-populism/. (Accessed April 9, 2020.)

[r7] Bonikowski, Bart. 2017 “Ethno‐Nationalist Populism and the Mobilization of Collective Resentment.” The British Journal of Sociology 68: 181–213. 10.1111/1468-4446.12325 29114869

[r8] Capoccia, Giovanni, and R. Daniel Kelemen. 2007 “The Study of Critical Junctures: Theory, Narrative, and Counterfactuals in Historical Institutionalism.” World Politics 59 (3): 341–369.

[r9] Castellanos-Jankiewicz, León. 2020 “COVID-19 Symposium: US Border Closure Breaches International Refugee Law.” OpinioJuris, April 3, 2020. http://opiniojuris.org/2020/04/03/covid-19-symposium-us-border-closure-breaches-international-refugee-law/. (Accessed April 9, 2020.)

[r10] Chin, Rita. 2017 The Crisis of Multiculturalism in Europe: A History. Princeton, NJ: Princeton University Press.

[r11] CNN.2020. “Photo Shows ‘Corona’ Crossed Out and Replaced with ‘Chinese’ in Trump’s Briefing Notes.” March 19, 2020 https://edition.cnn.com/world/live-news/coronavirus-outbreak-03-19-20-intl-hnk/h_21c623966aa148dbeed242de4e94943e. (Accessed April 9, 2020.)

[r12] Colborne, Michael. 2020 “As World Struggles to Stop Deaths, Far Right Celebrates COVID-19.” Al Jazeera, March 26, 2020. https://www.aljazeera.com/indepth/features/world-struggles-stop-deaths-celebrates-covid-19-200326165545387.html. (Accessed April 9, 2020.)

[r13] Cuerden, Gareth, and Colin Rogers. 2017. “Exploring Race Hate Crime Reporting in Wales Following Brexit.” Review of European Studies 9 (1): 158–164.

[r14] Daragahi, Borzou. 2020 “Coronavirus Could Be Used by Authoritarian Leaders as Excuse to Undermine Democracy, Experts Warn.” The Independent, March 17, 2020. https://www.independent.co.uk/news/health/coronavirus-us-cases-government-pandemic-democracy-covid-19-a9407011.html. (Accessed April 9, 2020.)

[r16] Dzankic, Jelena, and Lorenzo Piccoli. 2020 “Coronavirus: Citizenship Infected.” GlobalCit Blog, March 13, 2020. http://globalcit.eu/coronavirus-citizenship-infected/. (Accessed April 9, 2020.)

[r17] Eberhardt, Jennifer. 2019 Biased: Uncovering the Hidden Prejudice That Shapes What We See, Think, and Do. New York: Viking Books.

[r18] Edwards, Griffin Sims, and Stephen Rushin. 2018 “The Effect of President Trump’s Election on Hate Crimes.” SSRN, last modified January 31, 2019. https://ssrn.com/abstract=3102652. (Accessed April 9, 2020.)

[r19] Einstein, Albert. 1929 “What Life Means to Albert Einstein: An Interview by George Sylvester Viereck.” By George Sylvester Viereck. *The Saturday Evening Post*, October 26, 1929. https://www.saturdayeveningpost.com/wp-content/uploads/satevepost/what_life_means_to_einstein.pdf. (Accessed April 9, 2020.)

[r20] Erlanger, Steven. 2020 “The Coronavirus Inflicts Its Own Kind of Terror.” New York Times, April 6, 2020. https://www.nytimes.com/2020/04/06/world/europe/coronavirus-terrorism-threat-response.html. (Accessed April 9, 2020.)

[r21] Escobar, Natalie. 2020 “When Xenophobia Spreads Like A Virus.” *NPR Code Switch*, March 4, 2020. https://www.npr.org/2020/03/02/811363404/when-xenophobia-spreads-like-a-virus?t=1585605937840&t=1585738440331. (Accessed April 9, 2020.)

[r22] Euronews.2020 “Coronavirus: Portugal Grants Temporary Citizenship Rights to Migrants.” March 29, 2020. Available at https://www.euronews.com/2020/03/29/coronavirus-portugal-grants-temporary-citizenship-rights-to-migrants. (Accessed April 9, 2020.)

[r23] Farage, Nigel. 2020 “Coronavirus Has Shown We Are All Nationalists Now: Does Boris Johnson Realise That?” The Daily Telegraph, March 12, 2020. https://www.telegraph.co.uk/politics/2020/03/12/coronavirus-has-shown-nationalists-now-does-boris-johnson-realise/. (Accessed April 9, 2020.)

[r25] FRA (Fundamental Rights Agency). 2020 “Coronavirus Pandemic in the EU—Fundamental Rights Implications.” Bulletin 1. Luxembourg: Publications Office of the European Union https://fra.europa.eu/sites/default/files/fra_uploads/fra-2020-coronavirus-pandemic-eu-bulletin-1_en.pdf. (Accessed April 8, 2020.)

[r26] Friedman, Régine Mihal, and Gertrud Koch. 1989 “Juden-Ratten–Von der rassistischen metonymie zur tierischen Metapher in Fritz Hipplers Film Der Ewige Jude.” Frauen Und Film 47: 24–35.

[r72] Fritz Hippler (director), The Ewige Jude (The Eternal Jew), 1940.

[r27] Giles, Chris. 2020 “Global Economy Set for Sharpest Reversal Since Great Depression.” Financial Times April 3, 2020. https://www.ft.com/content/19d2e456-0943-42fc-9d2d-73318ee0f6ab. (Accessed April 9, 2020.)

[r28] Harari, Yuval Noah. 2020 “The World After Coronavirus.” Financial Times, March 19, 2020. https://www.ft.com/content/19d90308-6858-11ea-a3c9-1fe6fedcca75. (Accessed April 9, 2020.)

[r29] HCNM (High Commissioner on National Minorities). 2020 “OSCE High Commissioner on National Minorities Offers Recommendations on Short-Term Responses To COVID-19 That Support Social Cohesion.” Press Release, March 26, 2020. https://www.osce.org/hcnm/449170. (Accessed April 9, 2020.)

[r30] Hopkins, Valerie, and Ben Hall. 2020 “Chill Descends Upon Hungary After Viktor Orban’s Power-Grab.” Financial Times, April 3, 2020. https://www.ft.com/content/27243d36-bf9d-411f-89ed-1d118ae639f8. (Accessed April 9, 2020.)

[r31] Horowitz, Donald. L. 2001 The Deadly Ethnic Riot. Berkeley: University of California Press.

[r32] Inotai, Edit. 2020 “How Hungary’s Orban Blamed Migrants for Coronavirus.” EU Observer, March 20, 2020. https://euobserver.com/coronavirus/147813. (Accessed April 9, 2020.)

[r33] Jansen, Frank. 2020 “Wie Rechte das Coronavirus zur Hetze gegen Flüchtlinge benutzen.” Der Tagesspiegel, March 9, 2020. https://www.tagesspiegel.de/politik/video-mit-pistolenschuss-wie-rechte-das-coronavirus-zur-hetze-gegen-fluechtlinge-benutzen/25625008.html. (Accessed April 9, 2020.)

[r34] Jenne, Erin K. 2018 “Is Nationalism or Ethnopopulism on the Rise Today?” Ethnopolitics 17 (5): 546–552.

[r35] Jeung, Russel. 2020 *Incidents of Coronavirus Discrimination.* Report for A3PCON and CAA. http://www.asianpacificpolicyandplanningcouncil.org/wp-content/uploads/Stop_AAPI_Hate_Weekly_Report_4_3_20.pdf. (Accessed April 9, 2020.)

[r36] Johnson, Sam Fleming, and Guy Chazan. 2020 “Coronavirus: Is Europe Losing Italy?” Financial Times, April 6, 2020. https://www.ft.com/content/f21cf708-759e-11ea-ad98-044200cb277f. (Accessed April 8, 2020.)

[r37] Kazmin, Amy, Edward White, and Stefania Palma. 2020 “Muslims Fear Backlash of India’s Coronavirus Fury.” Financial Times, April 2, 2020. https://www.ft.com/content/33017d73-d526-42ea-a2ee-b09078684534. (Accessed April 9, 2020.)

[r38] Kelman, Ilan. 2011 Disaster Diplomacy: How Disasters Affect Peace and Conflict. London: Routledge.

[r39] Ker-Lindsay, James. 2000 “Greek‐Turkish Rapprochement: The Impact of Disaster ‘Diplomacy’?” Cambridge Review of International Affairs 14 (1): 215–232.

[r40] Kirschbaum, Erik, Laura King, and Meg Bernhard. 2020, “Nationalism Rears Its Head as Europe Battles Coronavirus With Border Controls.” LA Times, March 19, 2020. https://www.latimes.com/world-nation/story/2020-03-19/nationalism-could-rear-its-head-as-europe-battles-coronavirus. (Accessed April 9, 2020.)

[r41] Krastev, Ivan. 2020 “Seven Early Lessons From the Coronavirus, Views From the Council.” European Council on Foreign Relations, March 18, 2020. https://www.ecfr.eu/article/commentary_seven_early_lessons_from_the_coronavirus. (Accessed April 9, 2020.)

[r42] Malešević, Siniša. 2019 Grounded Nationalisms: A Sociological Analysis. Cambridge: Cambridge University Press.

[r43] Mudde, Cas. 2019 The Far Right Today. Cambridge: Polity Press.

[r44] Mudde, Cas. 2020 “Will the Coronavirus ‘Kill Populism’? Don’t Count On It.” The Guardian, March 27, 2020. https://www.theguardian.com/commentisfree/2020/mar/27/coronavirus-populism-trump-politics-response. (Accessed April 9, 2020.)

[r45] Nagle, Angela. 2017 Kill All Normies: Online Culture Wars from 4chan and Tumblr to Trump and the Alt-Right. London: Zer0 Books.

[r46] ODIHR (Office for Democratic Institutions and Human Rights). 2019 “Hate Crime Reporting 2018.” Hate Crime Reporting. https://hatecrime.osce.org. (Accessed April 9, 2020.)

[r47] OHCHR (Office of the United Nations High Commissioner for Human Rights).2020 “COVID-19 Fears Should Not Be Exploited to Attack and Exclude Minorities – UN Expert.” March 30, 2020. https://www.ohchr.org/EN/NewsEvents/Pages/DisplayNews.aspx?NewsID=25757&LangID=E. (Accessed April 9, 2020.)

[r48] Ozkirimli, Umut. 2020 “Coronationalism?” OpenDemocracy, April 14, 2020. https://www.opendemocracy.net/en/can-europe-make-it/coronationalism. (Accessed April 25, 2020.)

[r49] Pew Research Center. 2020 “As Economic Concerns Recede, Environmental Protection Rises on the Public’s Policy Agenda.” February 13, 2020. https://www.people-press.org/2020/02/13/as-economic-concerns-recede-environmental-protection-rises-on-the-publics-policy-agenda/. (Accessed April 9, 2020.)

[r50] Politico.2020 “Poll of Polls.” https://www.politico.eu/europe-poll-of-polls/. (Accessed April 9, 2020.)

[r51] Rachman, Gideon. 2020 “Nationalism Is a Side Effect of the Coronavirus.” Financial Times, March 23 2020. https://www.ft.com/content/644fd920-6cea-11ea-9bca-bf503995cd6f. (Accessed April 6, 2020.)

[r52] Rantanen, Terhi. 2012 “In Nationalism We Trust?” In Aftermath: The Cultures of the Economic Crisis, edited by Manuel Castells, Joao Caraca, and Gustavo Cardoso, 132–151. Oxford: Oxford University Press.

[r53] Rees, Jonas H., Yann P. M. Rees, Jens H. Hellmann, and Andreas Zick. 2019 “Climate of Hate: Similar Correlates of Far Right Electoral Support and Right-Wing Hate Crimes in Germany.” Frontiers in Psychology 10: 2328. 31681118 10.3389/fpsyg.2019.02328 PMC6813724

[r54] Robb, Amanda. 2017 “Anatomy of a Fake News Scandal.” Rolling Stone, November 16, 2017. https://www.rollingstone.com/politics/politics-news/anatomy-of-a-fake-news-scandal-125877/. (Accessed April 6, 2020.)

[r55] Robertson, Derek. 2018 “How an Obscure Conservative Theory Became the Trump Era’s Go-To Nerd Phrase.” Politico, February 25, 2018. https://www.politico.com/magazine/story/2018/02/25/overton-window-explained-definition-meaning-217010. (Accessed April 9, 2020.)

[r56] Rogers, Katie, Lara Jakes, and Ana Swanson. 2020 “Trump Defends Using ‘Chinese Virus’ Label, Ignoring Growing Criticism.” New York Times, March 18, 2020. https://www.nytimes.com/2020/03/18/us/politics/china-virus.html. (Accessed April 9, 2020.)

[r57] Sharpe, Matthew. 2017 “The Long Game of the European New Right.” The Conversation, March 23, 2017. http://theconversation.com/the-long-game-of-the-european-new-right-75078. (Accessed April 9, 2020.)

[r58] Slettebak, Rune T. 2012 “Don’t Blame the Weather! Climate-Related Natural Disasters and Civil Conflict.” Journal of Peace Research 49 (1): 163–176.

[r59] Snyder, Timothy. 2017 “The Reichstag Warning.” New York Review of Books Daily, February 26, 2017. https://www.nybooks.com/daily/2017/02/26/reichstag-fire-manipulating-terror-to-end-democracy/. (Accessed April 9, 2020.)

[r60] Speit, Andreas. 2017 “Reichsbürger – eine facettenreiche, gefährliche Bewegung” In Reichsbürger: Die unterschätzte Gefahr*,* edited by Andreas Speit, 7–21. Berlin: Christoph Links Verlag.

[r61] Spinney, Laura. 2017 Pale Rider: The Spanish Flu of 1918 and How It Changed the World. New York: PublicAffairs.

[r62] Stojanovic, Milica. 2020 “Serbia Pins Coronavirus Blame on Returning Serbs ‘Concealing Infection,’ ” *Balkan Insight*, April 3, 202. https://balkaninsight.com/2020/04/03/serbia-pins-coronavirus-blame-on-returning-serbs-concealing-infection/. (Accessed April 9, 2020.)

[r63] Strittmatter, Kai. 2020 “Däne werden geht im Moment nicht.“ Süddeutsche Zeitung, March 8, 2020. https://www.sueddeutsche.de/politik/daenemark-corona-virus-einbuergerung-1.4836462. (Accessed April 9, 2020.)

[r64] Sunstein, Cas R., and Adrian Vermeule. 2009 “Conspiracy Theories: Causes and Cures.” Journal of Political Philosophy 17 (2): 202–227.

[r71] Tisdall, Simon. 2020 “Power, equality, nationalism: how the pandemic will reshape the world,” The Observer, March 28, 2020 https://www.theguardian.com/world/2020/mar/28/power-equality-nationalism-howthe-pandemic-will-reshape-the-world. (Accessed April 9, 2020.)

[r65] Trump, Donald J. (@realDonaldTrump). 2020 “THIS IS WHY WE NEED BORDERS!” Twitter, March 23, 2020, 7:16 a.m. https://twitter.com/realDonaldTrump/status/1242092738973249536. (Accessed April 9, 2020.)

[r66] Waddy, Nicholas L. 2020 “Pandemic Nationalism.” American Greatness, March 23, 2020. https://amgreatness.com/2020/03/23/pandemic-nationalism/. (Accessed April 9, 2020.)

[r67] Wallace, Steven P., and Maria Elena de Trinidad Young. 2018 “Immigration Versus Immigrant: The Cycle of Anti-Immigrant Policies.” American Journal of Public Health 108 (4): 436–437. 29513594 10.2105/AJPH.2018.304328 PMC5844428

[r68] White, Johnathan. 2015 “Emergency Europe.” Political Studies 63 (2): 300–318.

[r69] Youde, Jeremy. 2020 “How ‘Medical Nationalism’ Is Undermining the Fight Against the Coronavirus Pandemic.” World Politics Review, March 23, 2020. https://www.worldpoliticsreview.com/articles/28623/how-medical-nationalism-is-undermining-the-fight-against-the-coronavirus-pandemic. (Accessed April 9, 2020.)

[r70] Zielger, Philip. 1998 The Black Death. 2nd ed. New York: Faber & Faber.

